# Detecting kelp-forest associated metazoan biodiversity with eDNA metabarcoding

**DOI:** 10.1038/s44185-023-00033-3

**Published:** 2024-02-21

**Authors:** Emma I. Rossouw, Jannes Landschoff, Andrew Ndhlovu, Götz Neef, Masaki Miya, Kira-Lee Courtaillac, Rouane Brokensha, Sophie von der Heyden

**Affiliations:** 1 Department of Botany and Zoology, Stellenbosch University, Private Bag X1, Matieland, 7602 South Africa; 2 Sea Change Project, Sea Change Trust, 6 Buxton Avenue, Oranjezicht, 8001 Cape Town South Africa; 3 School of Climate Studies, Stellenbosch University, Private Bag X1, Matieland, 7602 South Africa; 4 Natural History Museum and Institute, Chiba, 955-2 Aoba-cho, Chuo-ku, Chiba 260-8682 Japan

**Keywords:** Ecological genetics, Molecular ecology, Genetic markers, Ocean sciences

## Abstract

Environmental DNA (eDNA) metabarcoding is a promising tool for monitoring marine biodiversity, but remains underutilised in Africa. In this study, we evaluated the ability of aquatic eDNA metabarcoding as a tool for detecting biodiversity associated with a South African kelp forest, an ecosystem that harbours high diversity of species, many of which are endemic, but are also sensitive to changing environmental conditions and anthropogenic pressures. Using fine-scale spatial (1 m and 8 m) and temporal (every four hours for 24 h) sampling of aquatic environmental DNA and targeting two gene regions (mtDNA COI and 12S rRNA), metabarcoding detected 880 OTUs representing 75 families in the broader metazoan community with 44 OTUs representing 24 fish families. We show extensive variability in the eDNA signal across space and time and did not recover significant spatio-temporal structure in OTU richness and community assemblages. Metabarcoding detected a broad range of taxonomic groups, including arthropods, ascidians, cnidarians, echinoderms, ctenophores, molluscs, polychaetes, ichthyofauna and sponges, as well as Placozoa, previously not reported from South Africa. Fewer than 3% of OTUs could be identified to species level using available databases (COI = 19 OTUs, 12S = 11 OTUs). Our study emphasizes that kelp-forest associated biodiversity in South Africa is understudied, but that with careful consideration for sampling design in combination with increased barcoding efforts and the construction of regional databases, eDNA metabarcoding will become a powerful biomonitoring tool of kelp-forest associated biodiversity.

## Introduction

Despite the well-acknowledged importance of coastal marine environments, there remain key knowledge gaps in species distributions and community structuring, impeding conservation and management efforts. At the core of this deficiency lies that many marine biodiversity monitoring efforts are limited by logistical challenges such as working along high-energy and difficult-to-access coastlines, and the often time-consuming and costly nature of traditional survey methods^1,2^. As such, assessing environmental change, particularly those driven by anthropogenic pressures quickly and accurately in coastal marine ecosystems is difficult and there exists a need for innovative technologies that will support knowledge discovery, and comprehensive conservation decision-making over large geographical areas^2,3^.

Kelp forest ecosystems are limited to cold, nutrient-rich water, in which they occupy rocky reefs in predominantly dynamic, wave-exposed nearshore environments at temperate to subpolar latitudes. Kelp forests across the globe are valued for their ability to create biogenic habitats supporting a vast range of marine biodiversity, act as ecosystem engineers and provide a range of ecosystem services highlighting their conservation importance^4^. As with many coastal ecosystems globally, kelp forests are subjected to anthropogenic pressures such as climate change that drive shifts in kelp distributions with major social and ecological impacts^5^. As such, it is important to document contemporary community structures and composition changes to track spatio-temporal dynamics induced by these pressures. However, kelp forests exemplify challenging coastal marine environments for surveying, given their distribution in wave-exposed and high energy coastlines, that leaves their contemporary associated biodiversity remarkably understudied.

**Fig. 1:**
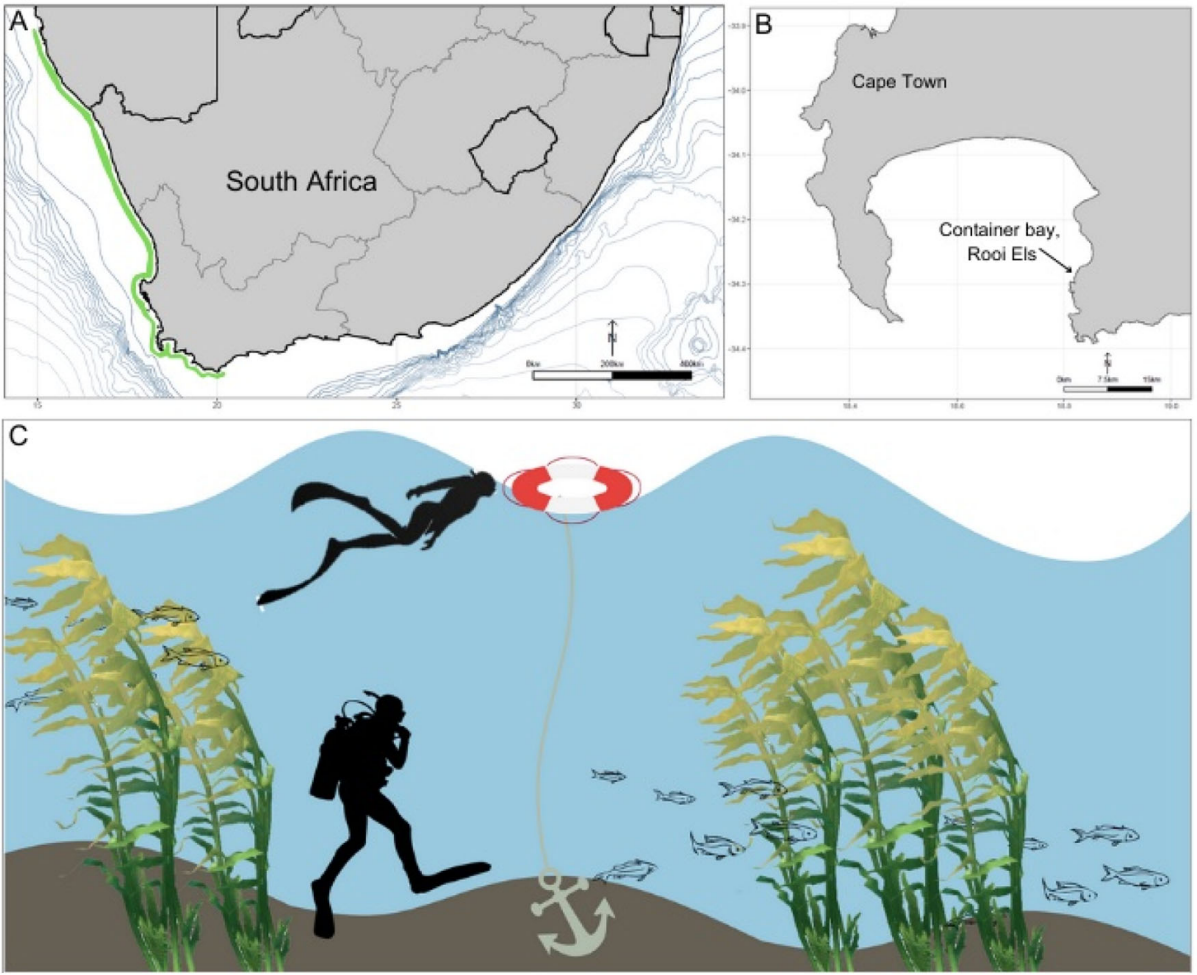
Overview of sampling of aquatic eDNA in a South African kelp forest. Extent of the Great African Seaforest along the southern African coast shown as a green line (**A**) and the sampling site of Container Bay, a +−150 m^2^ wave exposed sub-bay in False Bay (**B**). Water sampling at 1 m and 8 m depth was carried out by two divers concurrently, every four hours for 24 h (**C**).

In southern Africa, kelp forest ecosystems, informally known as the Great African Seaforest, harbour diverse marine communities^6^. The Great African Seaforest fringes southern Africa’s southwestern coastline, extending from Cape Agulhas in South Africa to ∼1000 km north into Namibia (Fig. 1), and grows primarily in high-energy and wave-exposed coastlines. The highly productive and tall growing kelps, dominated by *Ecklonia maxima* and *Laminaria pallida*, enhance biodiversity through a three-dimensional structure that provides habitat, nursery and shelter for a range of species, many of which are endemic to the region^7^. In contrast to other marine ecosystems, including kelp systems elsewhere that are experiencing declines^8^, the Great African Seaforest is extending its range eastwards^5^, due to cooling environments on the west and south-west coast. Nonetheless, kelp forests in South Africa are increasingly exposed to the impacts of anthropogenic pressures^9^, threatening their long-term persistence and ecosystem services, that have been estimated at ~5 billion rand (ZAR) per year^10^. Although South African kelp-forest associated biodiversity was extensively studied from the 1970s until the late 2000s^6,7,11,12^, there is great potential for marine biodiversity research in South Africa due to the anticipated large numbers of undiscovered species^7,13^. In addition, the increasing exposure to anthropogenic pressures^9^ requires a more comprehensive and contemporary understanding of kelp-forest associated biodiversity and how ecosystems and their components are changing.

Environmental DNA metabarcoding is a valuable tool for detecting contemporary marine biodiversity in that it can non-invasively detect population structure^14^, rare or threatened species^15^, and community composition^16^, aiding conservation management^17^. However, the reliability and resolution of eDNA assessments to capture contemporary marine communities is dependent on delineating the influence of multiple biotic (e.g., microbial activity) and abiotic (e.g. temperature, UV, tides, salinity) factors on the persistence and dispersal of eDNA signals^3,18,19^.

Although eDNA assessments are likely to vary with sampling temporality, previous studies conducted in coastal waters suggest that communities detected through eDNA reflect community assemblages over small spatio-temporal scales^20–23^, highlighting the localised nature of eDNA signals. Studies carried out in dynamic habitats such as coastlines with high wave-energy have demonstrated the applicability of using eDNA metabarcoding for assessing kelp-forest associated biodiversity^24–29^. Despite the dynamic nature of these study systems, eDNA signals were highly localised, rather than showing homogenous signals (likely because of water movement and mixing), highlighting the ability of eDNA to accurately describe communities spatially. Furthermore, eDNA assessments successfully detected a broad range of marine biodiversity providing more comprehensive species inventories for marine ecosystem monitoring.

In general, eDNA metabarcoding for biomonitoring remains under-utilised in Africa, with a lack of studies from marine systems^30^, although studies suggest unique community assemblages in near-shore environments^16^. Here, we utilise eDNA metabarcoding to detect two distinct communities: marine fishes and the broader metazoan community, to evaluate the potential of eDNA metabarcoding to detect regional kelp-forest associated taxa. By sampling at two depths and across a 24-hour period, we also intended to better understand the spatio-temporal variability of eDNA signals. As such, our study contributes to developing sampling designs to support regional biodiversity monitoring efforts.

## Materials and methods

### Study site and sample collection

Surveys were conducted at Rooiels, Western Cape, South Africa (34° 18’ 0” S, 18° 49’ 0” E) (Fig. 1). The site of Container Bay is a ± 150 m^2^ wave-exposed sub-bay in False Bay of ~10 m depth with a mixed sandy and rocky bottom. The rocky reefs are dominated by the Bamboo kelp *Ecklonia maxima*. Sampling was carried out over a 24-hour period from 8 am on the 5th May until 8 am on the 6th May 2022 (Supplementary Table S1). Swell ranged between 1.6–2 m, with the water temperature constant at 15 °C throughout sampling. High tide was at 05:18 and 17:51, low tide at 11:31 and 23:47 on the 5th May. On the 6th May, the high tide was at 05:52.

Every four hours, three replicates of 1 litre of water each were collected at both 1 and 8 m depth (in total *n* = 6 per time point), from the same site, which was pre-marked with a buoy (Fig. 1). Water samples were collected using bottles pre-sterilised in 10% sodium hypochlorite; the bottles were briefly rinsed in seawater prior to collection. Once onshore the 1 l samples were immediately passed through individual Sterivex™ (0.22 μm) filters using a sterile 50 ml syringe, with 2 ml ATL lysis buffer (Qiagen) added to each filter at the end of the filtration process before securing the filters with caps and parafilm. Additionally, three 500 ml field blanks (bottled mineral water) were filtered at 12:00 pm, 12:00 am and 04:00 am.

### DNA extraction, library preparation, sequencing and bioinformatic analyses

DNA was directly extracted from the Sterivex using the DNeasy Blood and Tissue kit (Qiagen), following a modified protocol^16^. Extractions were carried out in an ultra-clean, DNA-free room with surfaces sterilised through a combination of high intensity UV for 30 min, as well as frequent wiping with 10% sodium hypochlorite solution. To check for DNA contamination, DNA extractions were also performed on negative controls, which consisted of three field blanks and three lab blanks (of Ultra Clean DNA free water). DNA concentration of all extractions were quantified with a Qubit™ 1X dsDNA High Sensitivity Assay.

DNA replicates from each sampling time point were pooled prior to sequencing, resulting in a total of 16 samples and two blanks (one field and lab blank) used for library construction. As multi-primer approaches better capture community diversity^21^, we targeted both the mtDNA COI gene using the primers “mICOIintF” and “jgHCO2198” primer set^31^, as well as 12S rRNA gene using the MiFish-U/E primer set^32^ to specifically assess the kelp-forest associated metazoan community, as well as fishes respectively. Library preparation methods, sequencing, as well as bioinformatic analyses are provided in the Supplementary Methods S1 and S2. The final OTU filtering steps consisted of removing any operational taxonomic units (OTUs) that could not be verified by BOLD. Furthermore, OTUs that were not taxonomically assigned to marine metazoans were removed from the final dataset. Where possible, the taxonomic assignment of OTUs were checked against the primary literature, e.g. Smiths' Sea Fishes^33^. The final datasets with all OTUs (including blanks), taxonomic assignment as well as number of reads per sample can be accessed at www.github.com/vonderHeydenLab/Kelp-eDNA_Rossouw.

### Statistical analyses

Analyses were performed using the R package *vegan*^34^ in the R statistical environment (version 4.2.1), following Monuki et al.^27^ and carried out at the OTU level, to overcome the limitation of incomplete taxonomic association between genetic sequences and taxonomic identification. OTU accumulation curves were constructed with the *specaccum*^34^ function. To investigate whether eDNA signatures showed variation over time and depth, OTU richness, the total number of OTUs per sample was calculated. The OTU richness was compared with two-way ANOVAs, after the data was tested for the appropriate assumptions.

Permutational analyses of variance (PERMANOVA) were performed on Bray-Curtis dissimilarity distances with function the *adonis*^34^ function to determine whether the communities captured by each respective primer assays differed across sampling depth and sampling time. Because the interaction between time and depth was not significant, it was excluded from the model. To illustrate the temporal and spatial dynamics of eDNA signals, a heatmap and Venn diagram were constructed in *DisplayR*.

## Results

### Sequencing results

The number of reads ranged between 2 and 42,925 reads per sample for the COI and 95,326 and 180,429 for 12S (Supplementary Table S2). Of these, two samples of the COI dataset, T0 at 1 m and T6 at 1 m, did not have any reads and one sample in the 12S dataset only resolved five OTUs (T3 at 8 m). For the remainder of the samples and prior to filtering, we obtained a total of 167,740 reads belonging to 1290 operational taxonomic units (OTUs) for the COI dataset (12 samples), and 1,925,206 reads belonging to 68 OTUs for the 12S dataset (14 samples). The extraction controls and blanks (field and lab) had a negligible number of reads that were removed from the total dataset (0.2 ± 0.5 and 0.67 ± 0.46; mean ± *SE*). OTU accumulation curves for both the COI and 12S datasets revealed that the dataset (*n* = 14) was insufficient to comprehensively capture OTU diversity (Supplementary Fig. S1).

### OTU and taxonomic diversity across time and space

**Fig. 2:**
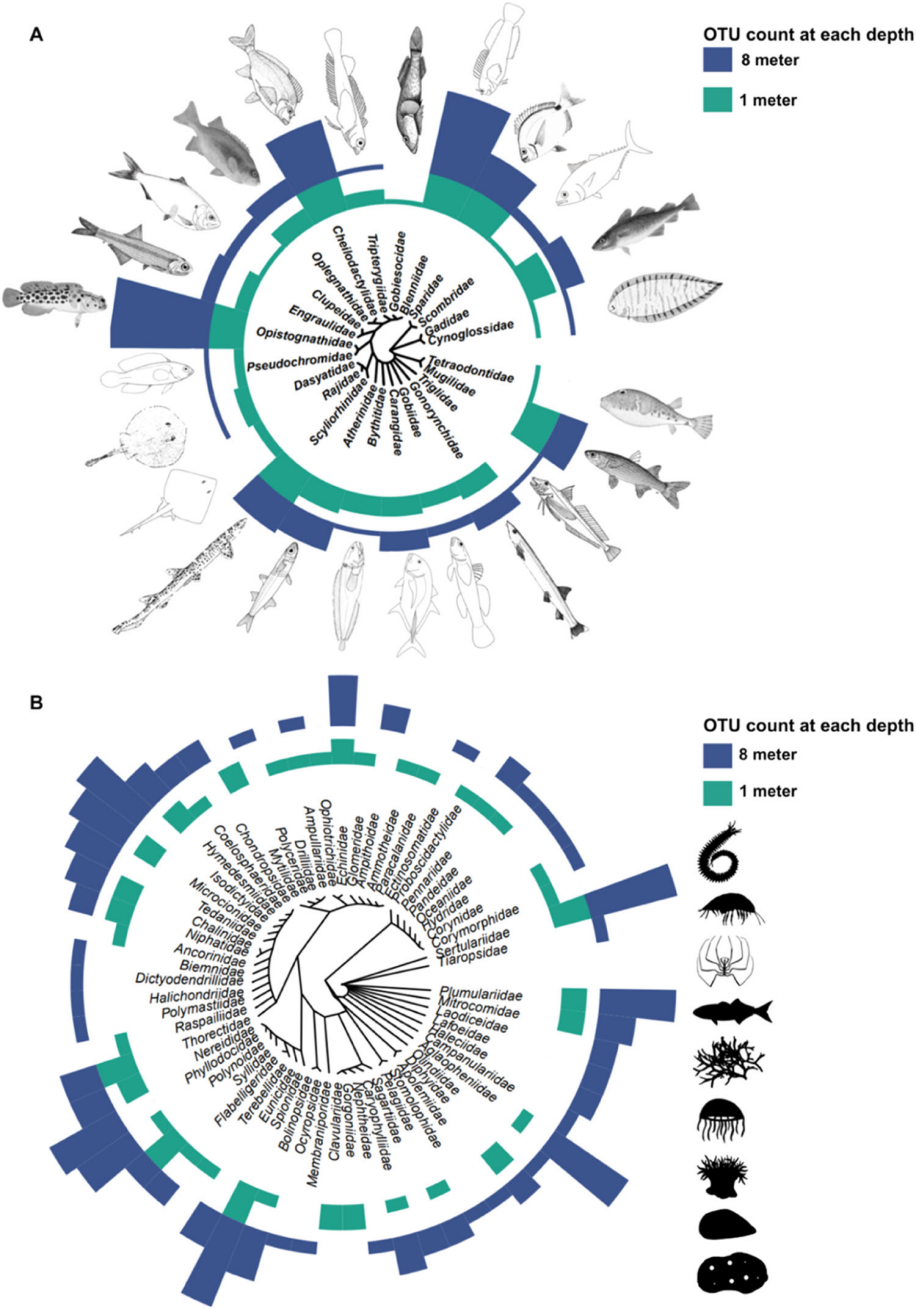
Dendrogram illustrating family level community assemblage and relative OTU abundance. **A** Fish community detected with 12S and **B** broader metazoan community detected with COI. Relative OTU abundance is provided by sampling depth.

Post filtering and after removal of non-eukaryotic and unicellular eukaryote OTUs, eDNA metabarcoding of the COI fragment, detected 880 metazoan OTUs, assigned across various taxonomic levels (Fig. 2), including 75 families, but with only 19 OTUs identified to species (Fig. 2). Some of the abundant taxonomic groups detected belonged to Cnidaria (104 OTUs), Arthropoda (43), Porifera (35), Annelida (33) and Mollusca (30). The least diverse groups were the Chaetognatha and Placozoa, both represented by one OTU. The major component in the Cnidaria was represented by Hydrozoa (84 OTUs) and for all cnidarians 93% were assigned to class, 76% to order, 37% to family, 14% to genus, with only 3% assigned to species. The second largest group detected was Arthropoda, of which 41% could be resolved to class, including Malacostraca, Hexanauplia, Ostracoda, Diplopoda and Pycnogonida. The major component of the Porifera (35 OTUs) was represented by the Demospongiae (32 OTUs). Of the 35 OTUs for Porifera, 94% were assigned to class, 80% to order, 65% to family, 51% to genus and 20% to species. Annelida were represented only by the polychaetes and included orders such as Phyllodocida, Terebellida, Eunicida and Spionida. Of the 9 OTUs assigned to genus level (27%) and only one was assigned to species (*Dipolydora capensis*). Of the Mollusca all of the OTUs were resolved to class and consisted of Polyplacophora (least abundant, with 6% of OTUs), Gastropoda (most abundant at 19% of OTUs) and Bivalvia. Only two OTUs could be resolved to species, *Semimytilus algosus*, an invasive mussel and *Aulacomya atra*, a native mussel species. For fishes, eDNA metabarcoding detected 44 OTUs across 24 families and 11 species (Fig. 2). Only two species (*Chorismoschismus dentex* and *Cheilodactylus fasciatus*) were detected by both the COI and 12S primer sets.

**Fig. 3:**
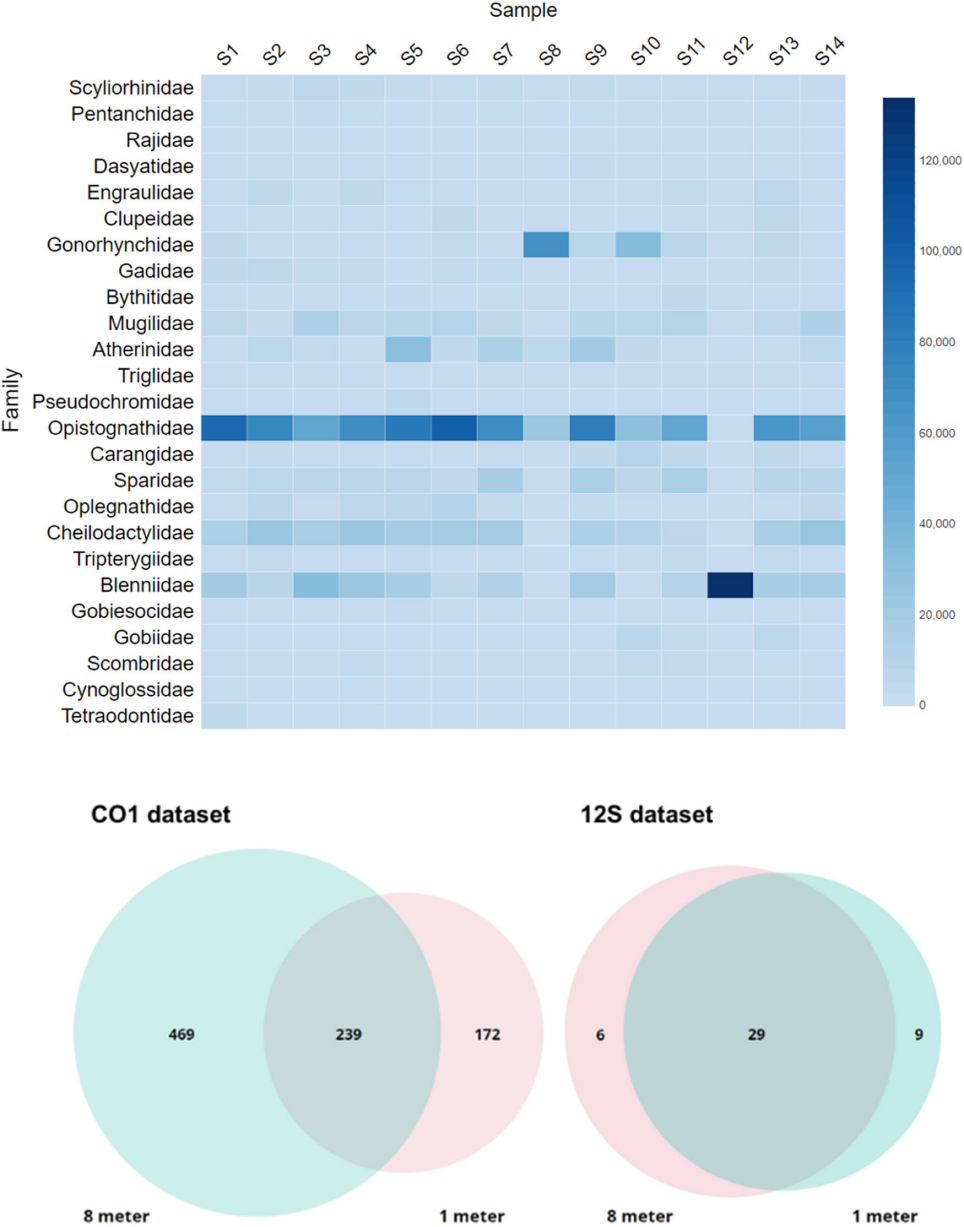
Diagrams illustrating spatio-temporal signals of eDNA across 24 h. **A** Heatmap showing the number of reads for each family of fishes by sample and **B** Venn diagram showing the number of OTUs recovered from the COI dataset and 12S dataset by depth. Sample information for the heatmap can be found in Supplementary Table S1.

There were no distinct differences in the eDNA signal across both space and time. A two-way ANOVA showed that OTU richness was not significantly different across time (COI: *F*_6_ = 0.414, *p* = 0.8462; 12S: *F*_6_ = 1.097, *p* = 0.457) or depth (COI: *F*_1_ = 4.638, *p* = 0.0747; 12S: *F*_1_ = 2.044, *p* = 0.203). Maximum and minimum OTU richness for both datasets were at T2 (16:00) and T3 (20:00) respectively. Overall, we detected almost three-fold the number of OTUs at 8 m compared to 1 m in the COI dataset, whereas OTUs detected for the 12 S data were more evenly (Fig. 3). The OTU read abundances of the 12S dataset exhibited heterogeneous patterns; for example, taxa such as Gonorhynchidae and Blenniidae were generally present at low detection levels, with a sudden spike of read abundances in samples S7 and S12 respectively, while an OTU belonging to Opistognathidaea was present at high detection levels throughout the samples (Fig. 3).

PERMANOVAs revealed that community composition did not significantly differ across depth (*F*_1_ = 0.93, *p* = 0.52) or time (*F*_6_ = 0.86, *p* = 0.80) for the COI dataset. Time of sampling accounted for most of the variation in community composition (*R*_2_ = 51%), with depth explaining the least (*R*_2_ = 9.2%). For the 12S dataset, community composition also did not significantly differ across depth (PERMANOVA; *F*_1_ = 1.36; *p* = 0.24) or time (PERMANOVA; *F*_6_ = 0.93; *p* = 0.57), with time explaining most of the variation (*R*^2^ = 43%) compared to depth (*R*^2^ = 11%).

## Discussion

Environmental DNA metabarcoding has transformed the monitoring of natural communities, including in dynamic marine environments. In this study, across 24 h and one sampling point in a South African kelp forest, we detected 880 OTUs for a broad diversity of marine eukaryotes and 44 OTUs specifically for ichthyofauna, providing further evidence of eDNA metabarcoding to support the biomonitoring of marine biodiversity in South Africa. This is despite the diversity uncovered being constrained by low read numbers for the COI dataset, which may not allow for the detection of rare species^35–37^, suggesting under-estimation of local kelp-associated biodiversity in this study. Nonetheless, metabarcoding detected a broad range of taxonomic groups, including arthropods, ascidians, cnidarians, echinoderms, ctenophores, molluscs, polychaetes, ichthyofauna, and sponges, as well as Placozoa which were previously not reported from South Africa^38^. However, there is limited literature providing species lists of kelp-forest associated biodiversity, from which to make comprehensive comparisons as most literature is either scattered across multiple sources or outdated^39^.

The most abundant taxonomic group detected during our eDNA survey was the Cnidaria, with Hydrozoa being the most abundant. These included genera with global distributions such as *Obelia* and *Plumularia* and species such as *Coryne eximia*. Besides benthic living colonies, eDNA also detected pelagic hydrozoa such as *Muggiaea*. Other macrobenthos detected include anthozoans such as the gorgon *Eunicella* and sea anemones including *Sagartia*, thus capturing a broad representation of cnidarian diversity.

The second most abundant group detected was the Crustacea, with most of the crustaceans captured by eDNA metabarcoding including very small and difficult to detect taxa such as amphipods and copepods (*Paracalanus, Oncaea* and *Ctenocalanus*). Environmental DNA metabarcoding also detected other arthropods including some pycnogonid taxa, which are relatively poorly studied. Annelid worms that are abundant throughout South African marine systems were represented by one class, the Polychaeta, with multiple families such as Flabelligeridae, Phyllodocidae and Syllidae, which are commonly found in kelp holdfasts (C. Katharoyan, pers. comm.).

Additional taxa detected include benthic animals such as the Cape urchin (*Parechinus angulosus*), a sea urchin endemic to southern Africa and a common species in intertidal and subtidal environments^6^. Despite the barcoding efforts of Sonet et al.^40^ that added 312 COI barcodes (primarily for species from the East coast of South Africa), many echinoderm OTUs could not be resolved to species level, including crinoids and brittlestars that are abundant at our sampling site and elsewhere in shallow water systems in South Africa. Interestingly, Sonet et al.^40^ showed that for almost 50% of morphospecies, there were significant discordances with their COI barcodes, suggesting a much higher echinoderm diversity than previously recognised. This is the scenario for many marine species in South Africa that likely contain cryptic diversity^7^ and thus the true biodiversity of South African marine systems, including those associated with kelp forest ecosystems, is likely to be much higher than currently recognised.

The MiFish primers detected OTUs (44 OTUs) and included ichthyofauna across a wide range of functional groups, such as benthic (clingfishes, rays, shysharks and tonguefishes) and more wide-ranging pelagic species (sparids, tunas and anchovies), that would be challenging to detect during traditional kelp biodiversity surveys, such as when utilising transects. Our study also provides support for using multiple primer assays as COI detected the Onefin electric ray, *Narke capensis*, that was not recovered with the MiFish primers. Overall only two fish species were detected with both primer sets, demonstrating that multi-marker approaches are needed to provide the most comprehensive community assemblage^21^.

As with many eDNA biodiversity surveys globally^15,30,41,42^, the lack of barcodes in reference databases limited taxonomic identification. Of the 880 OTUs that were detected by COI metabarcoding, only 19 OTUs were assigned to species level, of which seven were sponges, for which there have been recent barcoding efforts^43^, followed by the polychaete worms. These two groups have received recent taxonomic description and revision, which included phylogenetic approaches and as such provided valuable barcoding information^43,44^. Despite their diversity in kelp forest ecosystems, groups such as molluscs and arthropods had low taxonomic resolution, requiring extensive taxonomic and barcoding efforts.

Of the ichthyofaunal community, 11 OTUs were assigned to species, highlighting that continued efforts for barcoding regional marine biodiversity are essential^30,42,45^, including for fishes that generally have incomplete 12S rRNA databases^16^. Region-specific barcoding efforts are especially important in systems that display high levels of endemism, particularly in a South African context, where ~36% of marine species are endemic^7,46^. The limited number of OTUs that were taxonomically assigned to species may also reflect the megadiversity harboured by kelp forests and provides an example of a system that is highly understudied in its total taxonomic extent of biodiversity. However, despite the limitation imposed by the lack of a regional reference database, in just 24 h, eDNA metabarcoding detected more biodiversity than traditional methods would have the power to^2^, including small, elusive and rare diversity such as Placozoa, that are generally not detected by traditional monitoring tools. As such, eDNA metabarcoding should be more widely applied for studies of marine biodiversity in South Africa, in particular to provide baseline diversity information that can help plug several shortfalls^47^ in our knowledge of large-scale biodiversity distributions.

To better understand the temporal and spatial signals of eDNA representing the ichthyofauna and broader metazoan communities in a dynamic inshore system, we collected samples at two depths (1 m and 8 m), every four hours for 24 h, to better understand fine-scale signals of eDNA. This is important, as this variability in eDNA signals may influence which taxa are detected in space and time, a crucial component of sampling design. Our results showed that there was large variability in both ichthyofaunal and the broader metazoan communities, with no obvious structure in OTU richness and community assemblage across space or time, in contrast to other eDNA surveys in kelp forest ecosystems^24–29^. For example, we detected sessile benthic taxa such as crinoids, bivalves and sponges in samples collected at 1 m, suggesting some mixing of the water column. For fishes, generally benthic species such as *Bathytoshia brevicaudata* and *Cynoglossus zanzibarensis* were also detected at 1 m. These findings may result from numerous sources, including the presence of pelagic larval stages, or the mixing of the water column, which may homogenise the eDNA signals through transport. Besides abiotic factors such as water movement and wave-action, which were significant during our sampling campaign, particularly after 16 h, biotic factors such as ecological behaviour could also drive the fluctuation in eDNA signals, as kelp fauna display diurnal, horizontal and vertical dispersal^39^. Interestingly, the COI and 12S markers showed different degrees of overlap when analysed by depth (Fig. 3), with less overlap in detected taxa at 1 m and 8 m, with almost three-fold more taxa detected at 8 m. In contrast, there was little differentiation in the ichthyofaunal communities by depth. Given the variability in eDNA signals detected in our study, even though not statistically significant, it is likely that once-off sampling, either at depth or at one time point will not fully capture marine communities and as such, multiple sampling events will increase the likelihood of better representing biodiversity in an area. This may differ between sites given differences in patterns of local biodiversity and species abundances, as well as oceanographic conditions (such as swell, wind etc) during sampling, which may require different sampling approaches such as replication and sample volume^3^.

In addition, it is unclear to what extent our OTU database represents eDNA that spills over from adjacent ecosystems such as the transport of larvae from pelagic species from outside of the sampling areas, although evidence suggests eDNA detection limits range from several tens to hundreds of metres^26,48^. Incorporating long term replication in the study design for eDNA assessment, similar to Monuki et al.^27^, or more frequent collection of eDNA, could potentially circumvent the effect of DNA persistence on community signals and allow for the detection of fine-scale temporal differences. Regardless, for South African nearshore biodiversity surveys our findings highlight that sampling design needs to consider potential variability of eDNA signals to best capture marine biodiversity^3^.

Overall our study provides evidence that biodiversity associated with kelp forest ecosystems in South Africa is highly understudied and that large knowledge gaps exist across much of the biodiversity in these shallow-water coastal ecosystems. Although in some taxonomic groups it was possible to resolve OTUs to species, such as for some sponges, many OTUs detected with eDNA metabarcoding were underrepresented in reference databases. As such, increasing barcoding efforts of species associated with South African kelp forests will provide higher levels of species-level resolution and allow for the development of, for example, ecological network analyses to better support conservation initiatives. Further, combining approaches of visual monitoring, for instance through dive or Baited Remote Underwater Video (BRUV) surveys, as well as incorporating different sample substrates (water and sediment), will likely provide the most comprehensive inventories of biodiversity. As suggested by Jensen et al. and Lamy et al. ^20,28^ we concur that sampling across several time and depth points will enhance the detection of marine communities, compared to single sampling efforts. Overall, our work contributes to developing sampling protocols for aquatic eDNA metabarcoding for one of the world’s marine biodiversity hotspots and contributes to strengthening and increasing uptake of eDNA metabarcoding as a biomonitoring tool in Africa.

### Supplementary information

Supplementary Materials

## Data Availability

All data has been made available through www.github.com/vonderHeydenLab/Kelp-eDNA_Rossouw.
