# Supplementary material for: Detecting kelp-forest associated metazoan biodiversity with eDNA metabarcoding
Source: NPJ Biodivers. 2024 Feb 21;3:4. doi: 10.1038/s44185-023-00033-3 (PMC11332002; doi:10.1038/s44185-023-00033-3)
Supplement: Supplementary file 1 — Supplementary Materials [file 44185_2023_33_MOESM1_ESM.docx]

**SUPPLEMENTARY MATERIALS**

**Detecting kelp-forest associated metazoan biodiversity with eDNA metabarcoding**

Emma I. Rossouw^1^, Jannes Landschoff^1,2^, Andrew Ndhlovu^1,3^, Götz Neef^1^, Masaki Miya^4^, Kira-Lee Courtaillac^1^, Rouane Brokensha^2^, Sophie von der Heyden^1,3^

^1^Department of Botany and Zoology, Stellenbosch University, Private Bag X1, Matieland, 7602, South Africa

^2^Sea Change Project, Sea Change Trust, 6 Buxton Avenue, Oranjezicht, 8001, Cape Town, South Africa

^3^ School of Climate Studies, Stellenbosch University, Private Bag X1, Matieland, 7602, South Africa

^4^Natural History Museum and Institute, Chiba, 955-2 Aoba-cho, Chuo-ku, Chiba 260-8682, Japan

**TABLES**

**Table S1**. Times and depths of sample collection. Sunrise and sunset were at 07:23 AM and 17:59 PM respectively on the 5^th^ of May. On the 6^th^ of May, sunrise was at 07:24 AM. High tide was at 05:18 and 17:51, low tide at 11:31 and 23:47 on the 5^th^ . On the 6^th^, high tide was at 05:52.

| **Date** | **Time** | **Time point** | **Sampling Depth (m)** | **# Replicates collected** | **Code for Figure 3** |
| --- | --- | --- | --- | --- | --- |
| 05/05/2022 | 08:00 | T0 | 1 m | 3 | S1 |
|  |  |  | 8 m | 3 | S2 |
| 05/05/2022 | 12:00 | T1 | 1 m | 3 | S3 |
|  |  |  | 8 m | 3 | S4 |
| 05/05/2022 | 16:00 | T2 | 1 m | 3 | S5 |
|  |  |  | 8 m | 3 | S6 |
| 05/05/2022 | 20:00 | T3 | 1 m | 3 | S7 |
|  |  |  | 8 m | 3 | S8 |
| 06/05/2022 | 00:00 | T4 | 1 m | 3 | S9 |
|  |  |  | 8 m | 3 | S10 |
| 06/05/2022 | 04:00 | T5 | 1 m | 3 | S11 |
|  |  |  | 8 m | 3 | S12 |
| 06/05/2022 | 08:00 | T6 | 1 m | 3 | S13 |
|  |  |  | 8 m | 3 | S14 |

**Table S2.** Summary table for kelp forest eDNA sampling showing total number of sequencing reads and operational taxonomic units (OTUs) for each primer assay.

|  | **Pre-filtering** |  | **Post-filtering** | |
| --- | --- | --- | --- | --- |
| **Primer Assay** | **# Reads** | **# OTUs** | **# Reads** | **# OTUs** |
| CO1 (‘Leray’ primers) | 167,740 | 1290 | 88,555 | 880 |
| 12S (‘MiFish’ primer) | 1,925,206 | 68 | 1,884,119 | 44 |

**FIGURES**


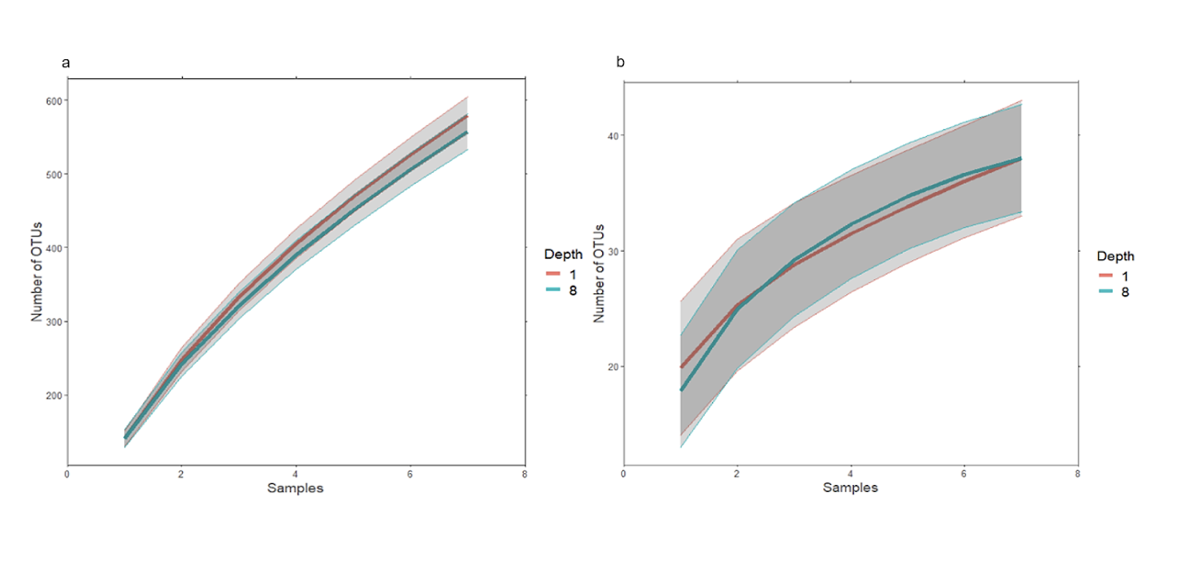


**Figure S1.** OTU accumulation curve of the (a) CO1 and (b) 12S datasets by each depth. Curves were constructed after removal of non-eukaryote and algal OTUs.

**METHODS**

**Method S1.** CO1 primer assay

1. Paired-end library preparation and sequencing

Amplification of the CO1-5P target region and preparation of the MiSeq libraries was performed with a 2-step PCR. A 313 bp long mini-barcode region was amplified by PCR following (Leray *et al.*, 2013; Morinière *et al.*, 2016), using forward and reverse HTS Leray primers, with complementary sites for the Illumina sequencing tails in triplicate. The PCR reaction was as follows: 95 °C for 5 min, 3 cycles of [96 °C for 15 s; 48 °C for 30 s; 65 °C for 90 s], 30 cycles of [96 °C for 15 s; 55 °C for 30 s; 65 °C for 90 s] and 76 °C for 10 min. The second PCR reaction used index primers with unique i5 and i7 inline tags and sequencing tails for amplification of indexed amplicons, applying the same annealing temperature as for the first PCR reaction but with only 7 PCR cycles. Equimolar amplicon pools were made, and size selected using preparative gel electrophoresis. MagSi-NGSprep Plus beads (Steinbrenner Laborsysteme GmbH, Wiesenbach, Germany) were used to purify the pooled DNA. A bioanalyzer (High Sensitivity DNA Kit, Agilent Technologies) was used to check the bp distribution and concentration of the amplicons before the creation of the final library. High-throughput sequencing (HTS) was performed on an Illumina MiSeq using v2 (2*250 bp, 500 cycles, maximum of 20mio reads) chemistry (Illumina).

1. Data preprocessing and taxonomic assignment

Bioinformatic analyses were performed by AIM Lab and following this protocol; raw FASTQ files from Illumina were processed using the VSEARCH suite v2.9.1 ^(Rognes^ *^et al.^*^, 2016)^ and Cutadapt v1.18 (Martin, 2011). Forward and reverse reads for each sample were merged with the VSEARCH program “fastq_mergepairs” and a minimum overlap of 10 bp, presenting sequences of ~313 bp. Forward and reverse primers were removed using Cutadapt 4.1 with Python 3.9.12. To discard sequences for which primers were not consistently detected at a minimum 90% identity, the “discard_untrimmed” option was used. Quality filtering with the “fastq_filter” in VSEARCH was used to keep sequences with zero expected errors (“fastq_maxee”). Sequences were dereplicated with “derep_fulllength,” at the sample level, and then concatenated into one FASTA file, which was dereplicated. The VSEARCH program “uchime_denovo” was used to filter out chimeric sequences from the FASTA file. The remaining sequences were clustered into OTUs at 97% identity with a greedy centroid-based clustering program, “cluster_size”. OTUs were blasted against databases (BOLD, NCBI and RDP) in February 2021, including taxonomy and BIN information, with Geneious (v.10.2.5—Biomatters, Auckland—New Zealand), and following the methods of ^(Morinière^ *^et al.^*^, 2016)^. The resulting csv file which included the OTU ID, BOLD Process ID, BIN, Hit%-ID value (percentage of overlap similarity- i.e., identical basepairs- of an OTU query sequence with its closest match in the database), length of the top BLAST hit sequence, phylum, class, order, family, genus, and species information for each detected OTU was exported from Geneious and combined with the OTU Table generated by the bioinformatic pipeline. The results were filtered by Hit-%-ID value and total read numbers per OTU. Entries with identifications below 97% and total read numbers below 0.01% of the summed reads per sample were removed from the analysis. OTUs were then assigned to the respective BIN. Additionally, the API provided by BOLD was used to retrieve BIN species and BIN countries for every OTU, and the Hit-%-IDs were aggregated over OTUs that found a hit in the same BIN and shown in the corresponding column as % range. To validate the BOLD BLAST results, a separate BLAST search was carried out in Geneious (using the same parameters) against a local copy of the NCBI nucleotide database downloaded from (ftp://ftp.ncbi.nlm.nih.gov/blast 16/db/). Species identification was based on high-throughput sequencing (HTS) data grouped to genetic clusters (OTUs), blasted, and assigned to barcode index numbers (“BINs”) ^(Ratnasingham & Hebert, 2013)^ which are a good proxy for species numbers ^(Ratnasingham & Hebert, 2013; Hausmann^ *^et al.^*^, 2013)^.

REFERENCES

Hausmann, A, Charles H, Godfray J, Huemer P, Mutanen M, Rougerie R, van Nieukerken EJ, Ratnasingham S, Hebert PDN (2013) Genetic patterns in European geometrid moths revealed by the Barcode Index Number (BIN) system. PLoS One 8: e84518. https://doi.org/10.1371/journal.pone.0084518.

Leray M, Yang JY, Meyer CP, Mills SC, Agudelo N, Ranwez V, Boehm JT, Machida RJ (2013) A new versatile primer set targeting a short fragment of the mitochondrial COI region for metabarcoding metazoan diversity: Application for characterizing coral reef fish gut contents. Frontiers in Zoology 10: 1–14. https://doi.org/10.1186/1742-9994-10-34.

Martin M (2011) Cutadapt Removes Adapter Sequences From High-Throughput Sequencing Reads. EMBnet Journal 17: 10–12.

Morinière J, Cancian De Araujo B, Lam AW, Hausmann A, Balke M, Schmidt S, Hendrich L, Doczkal D, Fartmann B, Arvidsson S, Haszprunar G (2016) Species identification in malaise trap samples by DNA barcoding based on NGS technologies and a scoring matrix. PLoS One 11: e0155497. https://doi.org/10.1371/journal.pone.0155497.

Ratnasingham S, Hebert PDN (2013) A DNA-Based Registry for All Animal Species: The Barcode Index Number (BIN) System. PLoS One 8: e66213. https://doi.org/10.1371/journal.pone.0066213.

Rognes T, Flouri T, Nichols B, Quince C, Mahé F (2016) VSEARCH: A versatile open source tool for metagenomics. PeerJ: e2584. https://doi.org/10.7717/peerj.2584.

**Methods S2.** 12S primer assay

1. Paired-end library preparation and sequencing

The workspace and equipment in the pre-PCR area were thoroughly sterilized prior to the library preparation. Filtered pipette tips were used while performing the pre- and post-PCR manipulations in two separate rooms, in order to safeguard against cross-contamination.

This study employed two-step PCR for paired-end library preparation using the MiSeq platform (Illumina, CA, USA). In general, we followed the methods developed by Miya et al. (2015) and subsequently modified by Miya and Sado (2019). For the first round of PCR (1st PCR), a mixture of the following six primers were used: MiFish-U-forward (5´–ACA CTC TTT CCC TAC ACG ACG CTC TTC CGA TCT NNN NNN GTC GGT AAA ACT CGT GCC AGC–3´), MiFish-U-reverse (5´–GTG ACT GGA GTT CAG ACG TGT GCT CTT CCG ATC TNN NNN NCA TAG TGG GGT ATC TAA TCC CAG TTT G–3´), MiFish-E-forward-v2 (5´–ACA CTC TTT CCC TAC ACG ACG CTC TTC CGA TCT NNN NNN RGT TGG TAA ATC TCG TGC CAG C–3´), MiFish-E-reverse-v2 (5´–GTG ACT GGA GTT CAG ACG TGT GCT CTT CCG ATC TNN NNN NGC ATA GTG GGG TAT CTA ATC CTA GTT TG–3´), MiFish-U2-forward (5´–ACA CTC TTT CCC TAC ACG ACG CTC TTC CGA TCT NNN NNN GCC GGT AAA ACT CGT GCC–3´), and MiFish-U2-reverse (5´–GTG ACT GGA GTT CAG ACG TGT GCT CTT CCG ATC TNN NNN NCA TAG GAG GGT GTC TAA TCC CCG TTT G–3´). These primer pairs amplify a hypervariable region of the mitochondrial 12S rRNA gene (ca. 172 bp; hereafter called “MiFish sequence”) and append primer-binding sites (5´ ends of the sequences before six Ns) for sequencing at both ends of the amplicon. The six random bases (Ns) present in the middle of these primers enhanced cluster separation on the flow cells during initial base-call calibrations on the MiSeq platform.

The 1st PCR consisted of 35 cycles with a 12 µL reaction volume containing 6.0 µL 2 × KAPA HiFi HotStart ReadyMix (KAPA Biosystems, MA, USA), 2.8 µl of a mixture of the four MiFish primers in a volume ratio of 2:1:1 (U:E:U2 forward and reverse primers; 5 µM), 1.2 µL sterile distilled H2O and a 2.0 µL eDNA template. To minimize PCR dropouts during the 1st PCR (Doi et al. 2019, Miya et al. 2020), eight technical replicates were performed for the same eDNA template using a strip of eight tubes (200 µL). The thermal cycle profile after an initial 3 min denaturation at 95 °C was as follows: denaturation at 98 °C for 20 s, annealing at 65 °C for 15 s, and extension at 72 °C for 15 s, with a final extension at the same temperature for 5 min. A 1st PCR blank (1 B) was also prepared during this process, in addition to EB. However, the blank PCR was not performed with replication and only a single tube was used for each of the two blanks (EB and 1 B) to minimize cost.

After completing the 1st PCR, an equal volume of PCR products were pooled from each of the eight replicates in a single 1.5 mL tube. The pooled products were purified using a GeneRead Size Selection kit (Qiagen, Hilden, Germany) following the manufacturer’s GeneRead DNA Library Prep I Kit protocol. This protocol repeats the column purification twice to completely remove the adapter dimers and monomers. Subsequently, the purified target products (ca. 300 bp) were quantified using TapeStation 2200 (Agilent Technologies, Tokyo, Japan), diluted to 0.1 ng/µL using Milli Q water, and the diluted products were used as templates for the second round of PCR (2nd PCR). For the two blanks (EB, 1 B), the 1st PCR products were purified in the same manner, but the purified PCR products were not quantified. Instead, they were diluted according to an average dilution ratio for the positive samples, following which the diluted products were used as templates for the 2nd PCR.

For the 2nd PCR, the following two primers were used to append dual-index sequences (eight nucleotides indicated by Xs) and flow cell binding sites for the MiSeq platform (5´ ends of the sequences before eight Xs): 2nd-PCR-forward (5´–AAT GAT ACG GCG ACC ACC GAG ATC TAC ACX XXX XXX XAC ACT CTT TCC CTA CAC GAC GCT CTT CCG ATC T–3´); and 2nd-PCR-reverse (5´–CAA GCA GAA GAC GGC ATA CGA GAT XXX XXX XXG TGA CTG GAG TTC AGA CGT GTG CTC TTC CGA TCT–3´).

The 2nd PCR was performed over 10 cycles with a 15-µL reaction volume containing 7.5 µL 2 × KAPA HiFi HotStart ReadyMix, 0.9 µL each primer (5 µM), 3.9 µL sterile distilled H2O and 1.9 µL template (0.1 ng/µL with the exceptions of the three blanks). The thermal cycle profile after an initial 3 min denaturation at 95 °C was as follows: denaturation at 98 °C for 20 s, annealing and extension combined at 72 °C (shuttle PCR) for 15 s, with the final extension at the same temperature for 5 min. A 2nd PCR blank (2 B) was also prepared during this process, in addition to EB and 1 B. The six PCR products as well as the three blank samples from the 2nd PCR products were pooled along with other samples from different projects.

Subsequently, the pooled dual-indexed libraries were electrophoresed on a 2% E-Gel Size Select agarose gel (Invitrogen, CA, USA). The target amplicons (~ 370 bp) were excised by retrieving them from the recovery wells using a micropipette. The concentration of the size-selected libraries was measured using a Qubit dsDNA HS assay kit and a Qubit fluorometer (Life Technologies, CA, USA), diluted to 10.0 pM with HT1 buffer (Illumina, CA, USA), and sequenced on the MiSeq platform using a MiSeq v2 Reagent Kit for 2 × 150 bp PE (Illumina, CA, USA) following the manufacturer’s protocol.

1. Data preprocessing and taxonomic assignment

Data preprocessing and analysis of raw MiSeq reads from the MiSeq run were performed using PMiFish ver. 2.4 (https://github.com/rogotoh/PMiFish.git; Miya et al. 2020) according to the following steps:

1) Forward (R1) and reverse (R2) reads were merged by aligning the two reads using the fastq merge pairs command. During this process, low-quality tail reads with a cut-off threshold set at a quality (Phred) score of 2, too short reads (<100 bp) after tail trimming, and those paired reads with too many differences (>5 positions) in the aligned region (ca. 65 bp) were discarded; 2) primer sequences were removed from those merged reads using the fastx truncate command; 3) those reads without the primer sequences underwent quality filtering using the fastq filter command to remove low quality reads with an expected error rate of >1% and too short reads of <120 bp; 4) the preprocessed reads were dereplicated using the fastx uniques command and all singletons, doubletons and tripletons were removed from the subsequent analyses to avoid false positives following the recommendation by the author of the program (Edgar 2010); 5) the dereplicated reads without single- to tripletons were denoised using the unoise3 command to generate amplicon sequence variants (ASVs) that removed all putatively chimeric and erroneous sequences (Callahan et al. 2017); 66) the ASVs were rarefied to the minimum read number (67,618); and 7) finally, ASVs were subjected to taxon assignments to species names (associated with operational taxonomic units; OTUs) using the usearch global command with a sequence identity of >98.5% with the reference sequences (two nucleotide differences allowed) and a query coverage of ≥90%.

Those ASVs with the sequence identities of 80–98.5% were tentatively assigned “U98.5” labels before the corresponding species names with the highest identities (e.g., U98.5 *Pagrus major*) and they were subjected to clustering at the level of 0.985 using the cluster smallmem command. An incomplete reference database necessitates this clustering step, which enables the detection of multiple OTUs for identical species names. Such multiple OTUs were annotated with “gotu1, 2, 3…” and all of these outputs (OTUs plus U98.5 OTUs) were tabulated with read abundances. ASVs with sequence identities of <80% (saved as “no hit”) were excluded from the above taxon assignments and downstream analyses because all of them were found to be non-fish organisms. MiFish DB ver. 43 was used for taxon assignment, comprising 7973 species distributed across 464 families and 2675 genera.

To refine the above taxon assignments, family level phylogenies were reproduced from MiFish sequences from OTUs, U98.5 OTUs, and reference sequences (contained in the MiFish DB ver. 43) belonging to these families. For each family, representative sequences (most abundant reads) from OTUs and U98.5 OTUs were assembled, all reference sequences were added from that family, and saved in FASTA format. The combined FASTA-formatted sequences were subjected to multiple alignments using MAFFT 7 (Katoh and Standley 2013) with a default set of parameters. A neighbour-joining (NJ) tree was subsequently constructed with the aligned sequences in MEGA X (Stecher et al. 2020) using Kimura two-parameter distances. The distances were calculated using pairwise deletion of gaps and among-site rate variations modelled with gamma distributions (shape parameter = 1). Furthermore, bootstrap resampling (n = 100) was performed to estimate the statistical support for the internal branches of the NJ tree and midpoint rooting was performed on the resulting NJ tree.

A total of 67 family-level trees were visually inspected and taxon assignments were revised in the following manner. For those U98.5 OTUs placed within a monophyletic group consisting of a single genus, the unidentified OTUs were named after that genus, followed by “sp.” with sequential numbers (e.g., *Pagrus* sp. 1, sp. 2, sp. 3...). For the remaining OTUs ambiguously placed in the family-level tree, the unidentified OTUs were named after that family, followed by “sp.” with sequential numbers (e.g., Sparidae sp. 1, sp. 2, sp. 3...).

 REFERENCES

Boettiger C, Lang DT, Wainwright PC (2012) rfishbase: exploring, manipulating and visualizing FishBase data from R. Journal of Fish Biology 81: 2030–2039. https://doi.org/10.1111/j.1095-8649.2012.03464.x

Callahan BJ, McMurdie PJ, Holmes SP (2017) Exact sequence variants should replace operational taxonomic units in marker-gene data analysis. The ISME Journal 11: 2639–2643. https://doi.org/10.1038/ismej.2017.119

Edgar RC (2010) Search and clustering orders of magnitude faster than BLAST. Bioinformatics 26: 2460–2461. https://doi.org/10.1093/bioinformatics/btq461

Froese R, Pauly D (2019) FishBase. Available from: www.fishbase.org.

Katoh K, Standley DM (2013) MAFFT Multiple Sequence Alignment Software Version 7: Improvements in Performance and Usability. Molecular Biology and Evolution 30: 772–780. https://doi.org/10.1093/molbev/mst010

Minamoto T, Miya M, Sado T, Seino S, Doi H, Kondoh M, Nakamura K, Takahara T, Yamamoto S, Yamanaka H, Araki H, Iwasaki W, Kasai A, Masuda R, Uchii K (2020) An illustrated manual for environmental DNA research: Water sampling guidelines and experimental protocols. Environmental DNA. https://doi.org/10.1002/edn3.121

Miya M (2022) Environmental DNA metabarcoding: A novel method for biodiversity monitoring of marine fish communities. Annual Review of Marine Science 14: 161–185. https://doi.org/https://doi.org/10.1146/annurev-marine-041421- 082251

Miya M, Sado T (2019) Multiple species detection using MiFish primers. In: eDNA-Method-Standardization-Committee (Ed.), Environmental DNA Sampling and Experiment Manual Version 2.1. The eDNA Society, Otsu, Japan, 55–92.

Miya M, Gotoh RO, Sado T (2020) MiFish metabarcoding: a high-throughput approach for simultaneous detection of multiple fish species from environmental DNA and other samples. Fisheries Science 86: 939–970. https://doi.org/10.1007/s12562-020-01461-x

Miya M, Sato Y, Fukunaga T, Sado T, Poulsen JY, Sato K, Minamoto T, Yamamoto S, Yamanaka H, Araki H, Kondoh M, Iwasaki W (2015) MiFish, a set of universal PCR primers for metabarcoding environmental DNA from fishes: detection of more than 230 subtropical marine species. Royal Society Open Science 2: 150088. https://doi.org/10.1098/rsos.150088

Society T eDNA (2019) Environmental DNA sampling and experiment manual Version 2.1. Available from: http://ednas ociety.org/eDNA_man-ual_Eng_v2_1_3b.pdf.

Stecher G, Tamura K, Kumar S (2020) Molecular Evolutionary Genetics Analysis (MEGA) for macOS. Molecular Biology and Evolution 37: 1237–1239. https://doi.org/10.1093/molbev/msz312
